# Multiobjective Robust Design of the Double Wishbone Suspension System Based on Particle Swarm Optimization

**DOI:** 10.1155/2014/354857

**Published:** 2014-02-11

**Authors:** Xianfu Cheng, Yuqun Lin

**Affiliations:** School of Mechanical and Electronical Engineering, East China Jiaotong University, Nanchang 330013, China

## Abstract

The performance of the suspension system is one of the most important factors in the vehicle design. For the double wishbone suspension system, the conventional deterministic optimization does not consider any deviations of design parameters, so design sensitivity analysis and robust optimization design are proposed. In this study, the design parameters of the robust optimization are the positions of the key points, and the random factors are the uncertainties in manufacturing. A simplified model of the double wishbone suspension is established by software ADAMS. The sensitivity analysis is utilized to determine main design variables. Then, the simulation experiment is arranged and the Latin hypercube design is adopted to find the initial points. The Kriging model is employed for fitting the mean and variance of the quality characteristics according to the simulation results. Further, a particle swarm optimization method based on simple PSO is applied and the tradeoff between the mean and deviation of performance is made to solve the robust optimization problem of the double wishbone suspension system.

## 1. Introduction

Suspension used in an automobile is a system mediating the interface between the vehicle and the road, and the functions of it are related to a wide range of drivability such as handing ability, stability, and comfortability [1]. There are many different structures of vehicle suspension system according to the mechanical jointing pattern, the type of springs, the independence of the left and right wheels, and so forth, of which the independent double wishbone suspension is extensively used.

With reference to automobile suspension system, a number of researches have devoted considerable efforts to design optimization. Many important relationships have been highlighted among vehicle suspension parameters and suspension performance indices [2]. These researches can be classed into several aspects: (1) a single-objective optimization of separately only considering reducing the dynamic load of the tire on the road or smoothness [3]; (2) transforming the traditional multiobjective optimization problem into single-objective optimization problem through a mathematical transformation [4]; (3) using multiobjective and multidecision optimization of true sense of the decision making after the first optimization [5]; (4) carrying out the analyses of displacement, velocity, and acceleration for McPherson strut suspension system using displacement matrix [6]. The optimal design is a balance of the kinematics and compliance characteristics of the suspension system [7]. But these approaches are based on conventional deterministic optimization and do not consider any deviations of design parameters, such as manufacturing errors of parts, which may result in unreliability of design objectives and constraints, and the computation time of these approaches is enormous. Robust design is powerful and effective in helping manufacturers to design their products and process as well as to solve troublesome quality problems, ultimately leading to higher customer satisfaction and operational performance [7]. However, a comprehensive multi-objective and robust approach seems to little be addressed. Chun et al. studied optimal designs for suspension systems based on reliability analyses [8]. Choi et al. performed a reliability optimization with the single-loop single-variable method by using results of a deterministic optimization as initial values of reliability-based optimization using the finite difference design sensitivity [9].

Robust optimization design is essentially multiple objectives: (1) optimizing the mean of performance and (2) minimizing the variation of performance. Since performance variation is often minimized at the cost of sacrificing performance, a tradeoff between the aforementioned two aims is generally presented. The particle swarm optimization (PSO) approach has demonstrated its strength in various types of multiobjective optimization design, including vehicles, aircrafts, and manufacturing facilities [10]. So, this study presents a new optimal method—based on robust design and PSO for suspension system. The objectives are the toe angle and the lateral slip of the wheel grounding point and their variations of the double wishbone suspension system. The mean and variance models are established by the Kriging model, and then PSO is used to analyze the robust performance of the system. This may help the designers to identify layout of the suspension system and to develop the optimum design system of suspension.

**Figure 1:**
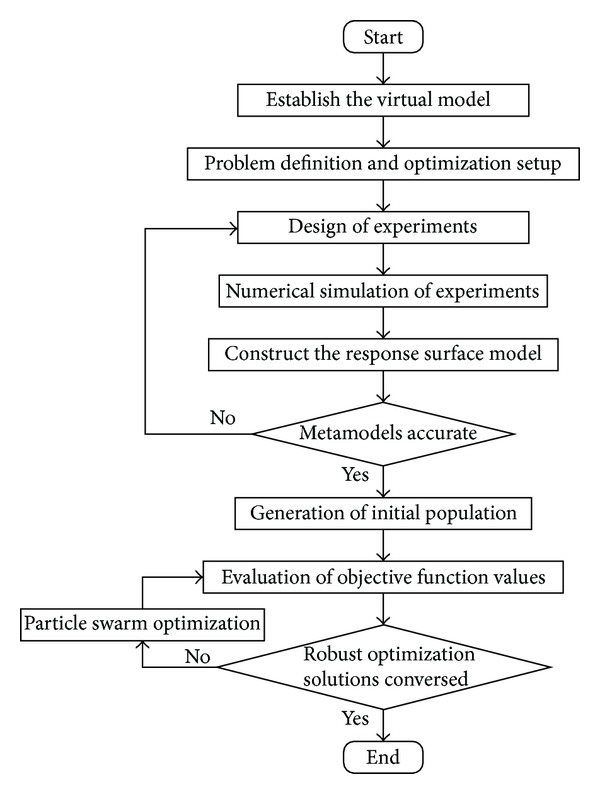
Robust design based on particle swarm optimization.

This paper is organized as follows: in Section 2, the virtual model of the double wishbone suspension system is established; in Section 3, the model of multi-objective robust optimization is built; the robust designs based on particle swarm optimization are described and analyzed in Section 4 and conclusions are presented in Section 5. The process of the robust design based on particle swarm optimization is shown in Figure 1.

## 2. The Virtual Model of the Double Wishbone Suspension System 

The double-wishbone suspension system is a group of space RSSR (revolute joint—spherical join—spherical join—revolute joint) four-bar linkage mechanisms. Its kinematics relations are complicated, kinematics visualization analysis is difficult, and its performance is poor. Thus, rational settings of the position parameters of the guiding mechanism are crucial to assuring good performance of the independent double-wishbone suspension.

**Figure 2:**
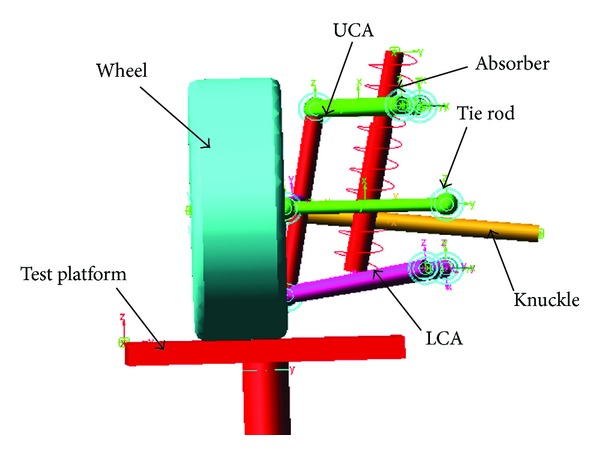
Double wishbone suspension model.

The vehicle's right and left suspensions are symmetrical, so choose the left or the right part of the suspension system which is studied to simulate the entire mechanism, excluding the variation of wheel centre distance (WCD) which is advisable. The key design parameters are the coordinates of the key points (see Table 1) and the assembly relationship between every member. A model of the left half of an independent double wishbone suspension system is established, as shown in Figure 2. Major components include the upper control arm (UCA), lower control arm (LCA), tie rod, knuckle, spring, and absorber. The design purpose of this study is to determine the positions of the joints. A commercial program, ADAMS, is employed for modelling and analysing the suspension system.

Make the following assumptions on double wishbone suspension.

1. The composition members of the suspension are rigid body, and the elastic deformation is ignored.
2. Rigid connection between the various components is used and ignores the internal clearance and friction.
3. Only consider the ground roughness, without regarding to the dynamic factors.
4. Add an incentive on the test platform to simulate the unevenness of the ground; the tires are always in contact with the test bench.

**Figure 3:**
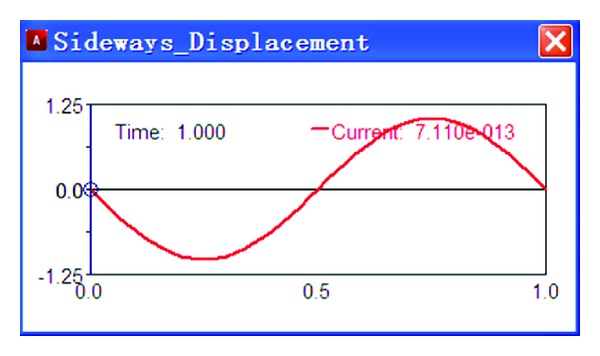
Sideways displacement.

**Figure 4:**
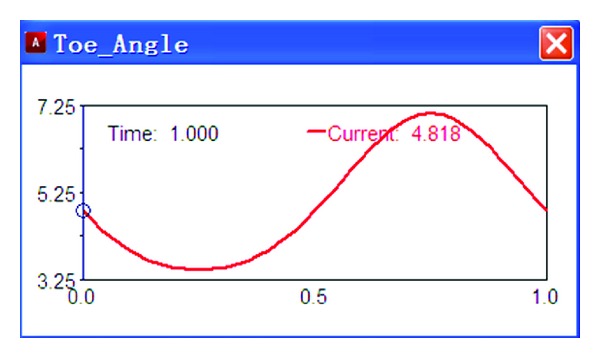
Toe angle.

Add an excitation source on the test platform, *y* = 50sin(2*πt*), and then taking numerical simulation, the results are shown in Figures 3 and 4.

As shown in Figure 3, the wheel sideways displacement changes with time. The change of sideways displacement is calculated according to the variation of the wheel travel. As shown in Figure 4, the toe angle changes with time. The change of toe angle is calculated according to the variation of the wheel travel too.

## 3. Model of the Robust Design and Approximation Model

In this section, the model of the robust design is built. A full-factor test and sensitivity analysis are utilized to determine main design variables. The Latin hypercube design is adopted to find the initial point, and the database is created for fitting the kriging model of the robust design.

### 3.1. Robust Design

Robust design has become a powerful tool to aid designers in making judicious selection and control of variation. The fundamental principle of robust design is to improve the quality of a product by eliminating the variation of controllable factors (i.e., dimension, assemble gap, material properties, etc.) and uncontrollable factors (i.e., applied loadings, environment, aging, etc.). Consequently, compared with traditional optimization design, robust design can make the product maintain good performance [11].

A standard engineering optimization problem is normally formulated as follows:

(1) min⁡⁡ f(x) s.t.   gj(x)≤0, j=1,2,…JxL<x<xU,

where *f*(*x*) is the objective function and *g*_*j*_(*x*) is the *j*th constraint function; *x*, *x*_*L*_, and *x*_*U*_ are vectors of design variables, their lower bounds, and upper bounds, respectively. If the design variable *x* follows a statistical distribution, a robust design problem can be stated as a biobjective robust design problem as follows:

(2) min⁡ ⁡[μf,σf] s.t.     gj(x)+kj∑i=1n|∂gj∂xi|Δxi, j=1,…,JxL+Δx≤x≤x  U  −Δx,

where *μ*_*f*_ and *σ*_*f*_ are the mean and deviation of the objective function *f*(*x*), respectively. Their values can be obtained through Monte Carlo simulation or the first order Taylor expansion if the design deviation of *x*_*i*_ is small. When using Taylor expansions, *μ*_*f*_ and *σ*_*f*_ can be represented by the following equations:

(3) $$\begin{array}{c} \mu_{f}=f\left(x\right),\\ \sigma_{f}^{2}={\overset{n}{\underset{i=1}{\sum }}\left(\frac{\partial_{f}}{\partial_{x_{i}}}\right)}^{2}\partial_{x_{i}}^{2}, \end{array}$$

where *σ*_*x*_*i*__ is the standard deviation of the *i*th *x* component.

### 3.2. Sensitivity Analysis

There are 12 key points, and each one of them has 3 coordinate values. So, there are 36 coordinate parameters. If every one of the coordinate is selected as design variables, it needs much iteration. In order to reduce time of analysis and save resources, the full-factor test is utilized to determine main design variable, and the impact of every dependent variable is in Table 2. There are three levels, 1–3, and the larger the value, the greater the impact of the dependent variable. As shown in Table 2, Lca front *x*, Lca outer *x*, Uca front *x*, Uca front *y*, Uca back *x*, and Uca outer *x* have made a minimal impact on sideways displacement and toe angle. Other coordinates of key points have made a great impact on sideways displacement and toe angle. Based on the test results, 12 main design variables are selected as controllable factors, and the variable name and its corresponding physical quantities are shown in Table 3.

### 3.3. Kriging Model

Engineering optimization problems often need enormous computation time for several programs running at the same time. We cannot provide the evaluation of the objective function and constraints to execute such large scale of exact analysis. So the application of approximation is necessary. In this paper, the Kriging model is adopted to build the approximation. Kriging model, one of the response surface models (RSM), has such advantages as unbiased estimator at the training sample point, desirably strong nonlinear approximating ability, and flexible parameter selection of the model, and thus it is quite suitable for approximate models [12]. Kriging models have a great promise for building accurate global approximations of a design space. These models are extremely flexible because of the wide range of spatial correlation functions that can be chosen for building the approximation, provided that sufficient sample data are available to capture the trends in the system responses; as a result, Kriging models can approach linear and nonlinear functions equally well. In addition, Kriging models can either “honor the data,” by providing an exact interpolation of the data, or “smooth the data,” by providing an inexact interpolation. One of the defects of using RSM in optimization is that it is apt to miss the global optimum because estimation value obtained with RSM includes errors at an unknown point [13].

In this paper the Kriging model is introduced into the robust design. In the conventional Kriging model, the performance *y*(*x*) is modelled as follows:

(4) $$\begin{array}{c} y\left(x\right)=\beta^{T}h\left(x\right)+Z\left(x\right), \end{array}$$

where *β* ^*T*^ *h*(*x*) is the regression component (e.g., a polynomial) which captures global trends; *Z*(*x*) is assumed to be a Gaussian process indexed by input variables *x*, with zero mean and stationary covariance.

From a Bayesian perspective, the prior knowledge of the performance *y*(*x*) is specified by a Gaussian process, which is characterized by the prior mean (i.e., the global trend) and prior covariance. Given the observations, the posterior process is also a Gaussian process (treating the covariance parameters as known and assuming a Gaussian prior distribution for *β*). The prediction of *y*(*x*) is usually taken to be the posterior mean, and the prediction uncertainty is quantified by the posterior covariance.

The conventional Kriging model assumes that the Gaussian process has a stationary covariance, with the covariance function defined as follows:

(5) $$\begin{array}{c} {\text{C}}_{\text{st}}\left(x_{m},x_{n};\Theta \right)=\sigma^{2}\rho_{\text{st}}\left(x_{m},x_{n};\theta \right), \end{array}$$

where *ρ*_st_ is the correlation function. The hyper parameter set Θ is composed of {*σ* ^2^; *θ*}. A frequently used Gaussian correlation function is

(6) $$\begin{array}{c} \rho_{\text{st}}\left(x_{m},x_{n};\theta \right)=\exp\left[-\overset{L}{\underset{L=1}{\sum }}\theta^{\left(l\right)}{\left(x_{m}^{\left(l\right)}-x_{n}^{\left(l\right)}\right)}^{2}\right]. \end{array}$$

The variance *σ* ^2^ provides the overall vertical scale relative to the mean of Gaussian process in the output space; *θ* = {*θ* ^(*l*)^ (*l* = 1,2,…, *L*)} are the correlation parameters (scaling factors) associated with each input variable *x* ^(*l*)^, which reflects the smoothness of the true performance. The stationary covariance indicates that the correlation function *ρ*_st_(*x*_*m*_, *x*_*n*_; *θ*) between any two sites *x*_*m*_ and *x*_*n*_ depends on only the distance (scaled by *θ*) between *x*_*m*_ and *x*_*n*_ in (5) and (6); the subscript “st” means “stationary.”

In order to innovate or improve and develop a new product and confirm a new technical parameter experiments usually need to be done repeatedly in the process of production and scientific research. It is very important to reasonably arrange experimental procedures to reduce the times of experiments and shorten the time of each experiment and avoid blindness. It requires two aspects of works to be done in order to solve the problem mentioned above. One is to design an experiment that can fully reflect the effect of all factors, which can reduce time of experiments and save resources. Another is to analyze the experimental results, in order to acquire reasonable conclusions and the error analysis.

DOE can analyze a design space and provide a rough estimate of an optimal design, which can be used as a starting point for numerical optimization. The Latin hypercube design could cover the design space more evenly than other DOE methods and generate more evenly distributed points. Therefore, in this paper, the Latin hypercube design is adopted to find the initial point and created the database for approximation model. For this problem, the inputs are the 12 main design variables, the outputs are the mean and their variance of the toe angle and sideways displacement, and 200 sample points from an LHS design are used to fit the kriging model. A set of 393 verification points, randomly selected across the domain, is used to evaluate the RMSE for each kriging model. Kriging model is established to fit the multiobjective robust design. The r-square of the kriging model is 0.87, so it can fit the virtual model.

## 4. Robust Design Based on Particle Swarm Optimization 

The particle swarm optimization (PSO) is one of the evolutionary computation techniques introduced by Kennedy and Eberhart in 1995 [14]. It is a population-based search algorithm and is initialized with a population of random solutions, named particles; PSO makes use of a velocity vector to update the current position of each particle in the swarm [15, 16].

Particle swarm optimization is usually used as a traditional optimization method which is inspired from the social behaviour of flocks of birds. It is more competitive in various aspects, for example, due to its simplicity. Particle swarm optimization, genetic algorithms, and other evolutionary algorithms are all artificial life calculated. But particle swarm optimization is different from other evolutionary algorithms, using group iterative solution of cooperation mechanisms to generate the optimal solution instead of using group iterative solution of competing mechanisms. In PSO algorithm, each individual is called “particle,” which represents a potential solution. The algorithm achieves the best solution by the variability of some particles in the tracing space. The particles search in the solution space following the best particle by changing their positions and the fitness frequently; the flying direction and velocity are determined by the objective function.

The procedure of PSO is as follows:

1. initialize the original position and velocity of particle swarm;
2. calculate the fitness value of each particle;
3. for each particle, compare the fitness value with the fitness value of pbest; if current value is better, then renew the position with current position, and update the fitness value simultaneously;
4. determine the best particle of group with the best fitness value; if the fitness value is better than the fitness value of gbest, then update the gbest and its fitness value with the position;
5. check the finalizing criterion; if it has been satisfied, quit the iteration; otherwise, return to step (2).

**Figure 5:**
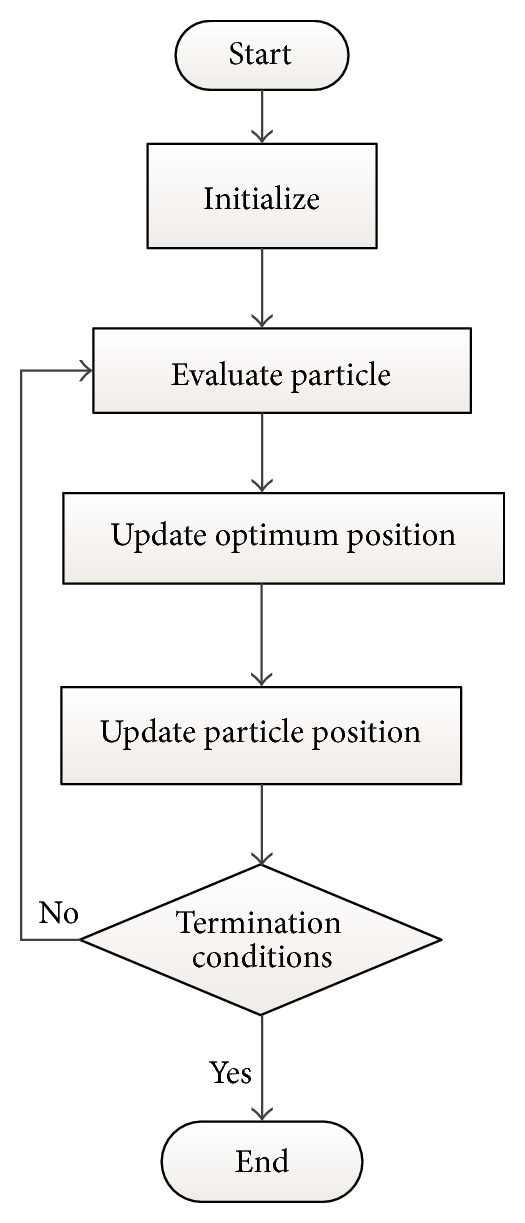
Particle swarm optimization.

It can be shown as Figure 5.

Assuming *X*_*i*_ = (*x*_*i*1_, *x*_*i*2_,…, *x*_*iD*_) is the position of *i*th particle in D-dimension, *V*_*i*_ = (*v*_*i*1_, *v*_*i*2_,…, *v*_*iD*_) is its velocity which represents its direction of searching. In iteration process, each particle keeps the best position pbest found by itself; besides, it also knows the best position gbest searched by the group particles and changes its velocity according to the two best positions. The PSO is described in vector notation as to the follows:

(7) νi(t+l)=ωνi(t)+c1r1(t)(pi(t)−xi(t))+c2r2(t)(pg(t)−xi(t)), i=1,2,…,s,xi(t+1)=xi(t)+j+νi(t+1),

where *s* is the swarm size. *c*_1_ and *c*_2_ are the nonnegative acceleration coefficients; these two constants make the particles have the ability of self-summary and learn from the excellent individuals of the groups, so the particles can close to the personal best solution of its own history and the global best solution within population or field. Typically value of *c*_1_ and *c*_2_ is 2. *ω* is the inertia weight, *r*_*l*_(*t*) and *r*_2_(*t*) ~ *U*(0, 1),  *x*_*i*_(*t*) is the position of particle *i* at time *t*, *ν*_*i*_(*t*) is the velocity of particle *i* at time *t*, *p*_*i*_(*t*) is the personal best solution of particle *i* at time *t*, and *pg*(*t*) is the global best solution at time *t*.

The first term of (8) is the previous velocity of the particle vector. The second and third terms are used to change the velocity of the particle. Without the second and third terms, the particle will keep on “flying” in the same direction until it hits the boundary. The particle position *x*(*t* + *l*) is updated using its current value and the newly computed velocity *v*_*i*_(*t* + *l*), which is determined by the values of *v*_*i*_(*t*), *x*_*i*_(*t*), *p*_*i*_(*t*), and *pg*(*t*) and coefficients *ω*, *c*_1_, and *c*_2_ [17].

In experiment, the population of group particle is 40; *c*_1_, and *c*_2_ are set to 2; the maximum time of iteration is 10000. It is acceptable if the difference between the best solution obtained by the optimization algorithm and the true solution is less than 1*e* − 6. The inertia weight is linear decreasing inertia all, which is determined by the following equation:

(8) $$\begin{array}{c} w=w_{\max}-\frac{w_{\max}-w_{\min}}{\text{ite}{\text{r}}_{\max}}\times k, \end{array}$$

where *w*_max⁡_ is the start of inertia weight which is set to 0.9 and *w*_min⁡_ is the end of inertia weight which is set to 0.05; iter_max⁡_ is the maximum times of iteration; *k* is the current iteration times. In order to reflect the universality of experiment, the original position and velocity are randomly generated.

Particle swarm optimization was used to search the optimal solution. A particle swarm optimization is created, the maximum iterations are set to 50, the number of particles is set to 15, and the objectives are the values and their variations of the toe angle and sideways displacement. V. Pareto, the French economist, who studied the multi-objective optimization problem of economics first, proposed the concept of Pareto solution set. There are 27 Pareto solutions in the results of the optimization.

**Figure 6:**
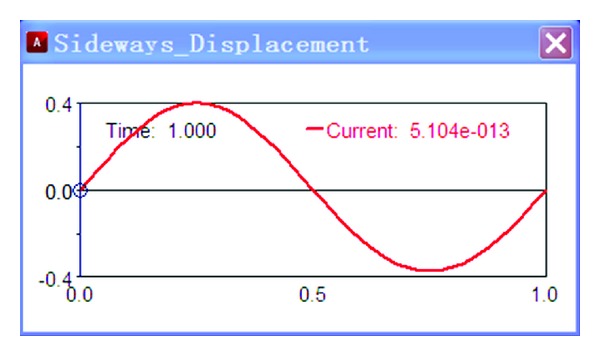
Sideways displacement.

**Figure 7:**
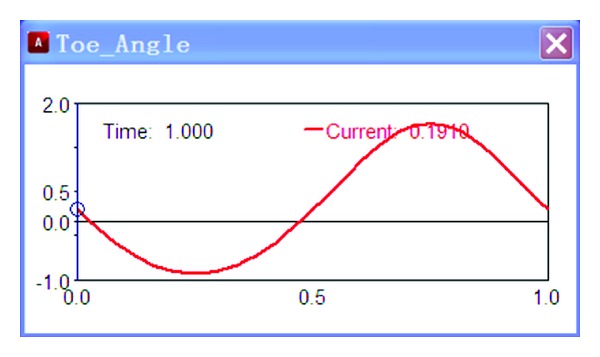
Toe angle.

In multiobjective optimization, each optimization objective is often conflicting, which requires coordination between the optimal solutions of each target. Considering the importance of each target, choose one Pareto optimal solution, and the design values of it are shown in Table 4. Using the results of the robust design to have a test in the ADAMS, the simulation results are shown in Figures 6 and 7.

It can be seen that the maximum deviation in the toe angle for the optimal design has been reduced by 52 percent, compared with the base design. As the discussion of the results, the most concerned factor is the relationship between objective function and design parameter. By comparing the experimental results, the robust design based on particle swarm significantly improved the robust of the toe angle and the sideways displacement, ensuring the reasonable of the design performance.

## 5. Conclusion 

In this study, a robust design based on bioinspired computation is presented and illustrated by the design of a double wishbone suspension system in order to reduce the effect of variations due to uncertainties in fabrication. As they are directly related to fabrication errors, the coordinates of key points were taken as design variables and at the same time are considered as random variables. So the robust design optimization problem had 13 design variables (joint positions) and 13 random constants (fabrication errors of joint positions). In this paper, the Latin hypercube design is adopted to make DOE design matrix of the 13 design variables. The Kriging model is built according to the result of DOE, and then the particle swarm is used to search optimal solution of the robust design. Particle swarm is implemented in a test case and the results show that the method can decrease the solution's time. The robustness of solution is improved. The improvement in robustness became larger as the amount of fabrication errors increases.

## Figures and Tables

**Table 1:** Key point.

Key point	*x*	*y*	*z*
Lca front	−200	−400	150
Lca back	200	−450	155
Lca outer	0	−750	100
Uca front	100	−450	525
Uca back	250	−490	530
Uca outer	40	−675	525
Shaft inner	0	−200	225
Spring lower	0	−600	150
Subframe front	−400	−450	150
Subframe back	400	−450	150
Tie rod inner	200	−400	300
Tie rod outer	150	−750	300
Spring upper	40	−500	650
The center of the wheel	0	−800	300

**Table 2:** Impact of each variable.

Coordinates of key points	Impact	
	Toe angle	Sideways displacement
Lca front *x*	1	1
Lca front *y*	2	2
Lca front *z*	3	3
Lca back *x*	1	2
Lca back *y*	1	3
Lca back *z*	2	3
Lca outer *x*	1	1
Lca outer *y*	1	3
Lca outer *z*	3	2
Uca front *x*	1	1
Uca front *y*	1	1
Uca front *z*	3	3
Uca back *x*	1	1
Uca back *y*	1	2
Uca back *z*	2	3
Uca outer *x*	1	1
Uca outer *y*	1	3
Uca outer *z*	3	3

**Table 3:** Controllable factors.

Key point	Level 1	Level 2	Level 3
Lca front *y*(*x*_1_)	−405	−400	−395
Lca front *z*(*x*_2_)	145	150	155
Lca back *x*(*x*_3_)	195	200	205
Lca back *y*(*x*_4_)	−455	−450	−445
Lca back *z*(*x*_5_)	150	155	160
Lca outer *y*(*x*_6_)	−755	−750	−745
Lca outer *z*(*x*_7_)	95	100	105
Uca front *z*(*x*_8_)	520	525	530
Uca back *y*(*x*_9_)	−495	−490	−485
Uca back *z*(*x*_10_)	525	530	535
Uca outer *y*(*x*_11_)	−680	−675	−670
Uca outer *z*(*x*_12_)	520	525	530

**Table 4:** Robust results.

Key point	Initial value	Robust results
Lca front *y*(*x*_1_)	−400	−400
Lca front *z*(*x*_2_)	150	150.33
Lca back *x*(*x*_3_)	200	205
Lca back *y*(*x*_4_)	−450	−450
Lca back *z*(*x*_5_)	155	151.67
Lca outer *y*(*x*_6_)	−750	−752.5
Lca outer *z*(*x*_7_)	100	101.33
Uca front *z*(*x*_8_)	525	519.67
Uca back *y*(*x*_9_)	−490	−489.67
Uca back *z*(*x*_10_)	530	532.17
Uca outer *y*(*x*_11_)	−675	−679.83
Uca outer *z*(*x*_12_)	525	523.33
